# Eating Disorder Symptoms in Multiple Sclerosis: Relationships Between Neuroticism, Body Dissatisfaction, and Self-Esteem

**DOI:** 10.3390/nu17101609

**Published:** 2025-05-08

**Authors:** Litza Kiropoulos, Isabel Krug, Phuong Linh Dang

**Affiliations:** 1 Mood and Anxiety Disorders Lab, Melbourne School of Psychological Sciences, University of Melbourne, Melbourne, VIC 3010, Australia; p.dang3@student.unimelb.edu.au; 2 Eating Disorders Lab, Melbourne School of Psychological Sciences, University of Melbourne, Melbourne, VIC 3010, Australia; isabel.krug@unimelb.edu.au

**Keywords:** eating disorder, neuroticism, body dissatisfaction, self-esteem, multiple sclerosis, depression, anxiety, neuroticism, serial mediation

## Abstract

**Background/Objectives**: Research on eating disorders (EDs) in individuals with multiple sclerosis (MS) is limited. In ED populations, neuroticism has been linked to ED symptoms through lower self-esteem and greater body dissatisfaction, but these relationships remain unexplored in MS. This study aimed to examine whether self-esteem and body dissatisfaction mediate the link between neuroticism and ED symptoms in individuals with MS. **Methods**: The current sample consisted of 275 participants who reported a neurologist-confirmed diagnosis of MS (*M*age = 43.0, SD = 12.9) with the majority being female (218/275; 79.3%). Participants completed an online questionnaire measuring neuroticism (Big Five Inventory), self-esteem (Rosenberg Self-esteem Scale), body dissatisfaction (Body Shape Questionnaire), and ED symptoms (Eating Attitudes Test-26). **Results**: A serial mediation analysis controlling for age, sex, and level of ambulation revealed that the association between neuroticism and ED symptoms is respectively and serially explained by self-esteem and body dissatisfaction in individuals with MS. The total model accounted for 43% of the variance in ED symptoms. **Conclusions**: Findings suggest that self-esteem and body dissatisfaction are important in understanding the relationship between neuroticism and ED symptoms. Neuroticism, self-esteem, and body dissatisfaction may be important targets for assessing and treating EDs in individuals with MS. Future longitudinal research is needed to examine causal relationships.

## 1. Introduction

Multiple sclerosis (MS) is a chronic autoimmune disease that affects the central nervous system, often leading to physical disability, cognitive difficulties, and emotional distress [1]. These psychological challenges may contribute to body image concerns and disordered eating (DE) behaviours, yet research on these symptoms in individuals with MS remains limited [2]. Eating disorders (EDs) are characterised by disturbances in eating behaviours and body image [3,4], with personality factors such as neuroticism playing a key role in their development and maintenance [5,6]. Higher levels of neuroticism have been linked to greater DE symptoms [7,8], body dissatisfaction [9,10], and self-esteem [11,12] in community and inpatient and outpatient ED populations. Several studies have demonstrated that self-esteem and body dissatisfaction function as mediating variables in the association between neuroticism and DE symptoms [13,14,15]. However, these associations remain unexplored in individuals with MS, despite the potential impact of MS-related physical and psychological changes on body image concerns and eating behaviours [16]. The present study, therefore, aimed to examine the relationships between neuroticism, self-esteem, body dissatisfaction, and DE symptoms in individuals with MS.

### 1.1. Eating Disorder Symptoms and Multiple Sclerosis

Although EDs have been widely studied in the general population [17] and among individuals with psychiatric disorders [18], research on body image concerns and DE symptoms in chronic illnesses such as MS remains limited. Chronic conditions that impact physical appearance and mobility have been linked to heightened vulnerability to body dissatisfaction and DE [19,20,21]. Emerging evidence suggests that DE may be more prevalent in individuals with chronic illnesses due to disease-related changes and their psychosocial consequences. For instance, conditions such as rheumatoid arthritis [19], diabetes [21], and cancer [20] have been associated with significant body image concerns, with studies indicating that DE behaviours in these populations may worsen over time.

Individuals with MS often experience symptoms such as fatigue, muscle weakness, motor impairments, and cognitive deficits, which can significantly impact their quality of life [1]. Beyond the physical manifestations of the disease, psychological factors such as depression, anxiety, and reduced self-esteem are highly prevalent in this population [22,23,24]. Emerging research suggests that body image disturbances and DE behaviours may also be concerns for individuals with MS, though this area remains underexplored [2,25,26,27]. It is possible that the presence of MS-related physical disabilities and weight fluctuations, as well as obesity, may contribute to concerns about body image, which in turn can influence eating behaviours. Corticosteroid treatments often prescribed for MS management are known to cause weight gain and changes in body composition, which may exacerbate body dissatisfaction and increase vulnerability to maladaptive eating behaviours [28]. Additionally, fatigue and mobility impairments may alter physical activity levels, leading to frustration with body changes and increased risk for DE patterns [29].

Research on the role of diet in the treatment of MS is also inconclusive, with variability in dietary recommendations. While some studies suggest that fat intake may worsen disease progression and vegetable consumption may be protective, a few meta-analyses indicated insufficient high-quality evidence to confirm the effectiveness of specific dietary interventions [30,31]. Such inconsistent findings may also partially cause body dissatisfaction and DE in several ways. First, the lack of clear dietary recommendations for MS may contribute to confusion and anxiety among patients, potentially leading to restrictive or DE behaviours to manage their symptoms. Patients may internalise messages about “good” and “bad” foods, increasing food-related distress and body dissatisfaction, particularly if weight gain or metabolic disorders associated with MS become a concern. Additionally, the emphasis on dietary control as a means of managing MS symptoms could heighten preoccupation with food and body image, further increasing the risk of DE patterns. Overall, the uncertainty surrounding dietary advice may leave patients vulnerable to misinformation, restrictive dieting, compensatory behaviours, or heightened body image concerns to exert control over their health.

Research on body image concerns in MS has also yielded mixed findings, highlighting the complexity of this issue [2,25,26,27]. Pfaffenberger et al. [27] found that individuals with MS reported greater concerns about physical deficits, poorer body appraisal, and more sexual difficulties compared to healthy controls, with notable gender-specific differences. Women with MS, in particular, predominantly struggled with feelings of unattractiveness and worries about physical deficits, while men with MS reported prevalent concerns regarding sexual dysfunction, independent of disease severity and depression symptoms [27]. This study established a foundational understanding of how MS-related physical changes may impact body image. Building on this, Bailey et al. [26] explored the broader sociocultural factors influencing body image among middle-to-older-aged women with MS, emphasising the interplay between ageing, disability, and societal beauty standards. Their findings suggest that body image concerns in MS are not only shaped by disease-related physical changes but also by external societal pressures (e.g., weight stigma, the expectation to maintain a youthful appearance, and other beauty discourses), often leading women to criticise their own body or body parts [26]. Further refining this understanding, Stevens et al. [2] examined body image dissatisfaction in MS and found that, while overall levels of body dissatisfaction were comparable to the general population, it was more pronounced in women and associated with higher body mass index (BMI), depression, and experiences of stigma although no differences were found for age. This study highlighted the need to consider individual differences such as gender, BMI, and age, particularly in relation to psychological distress and weight-related concerns when assessing body image in individuals with MS. In contrast, Reininghaus et al. [25] reported that MS patients experienced fewer sexual problems than normative body image concern values and maintained stable relationships, suggesting that body image concerns may not be universally heightened across all individuals with MS. These varying findings underscore the need for further research to clarify the conditions under which body image disturbances and DE emerge in MS.

### 1.2. The Role of Neuroticism in Eating Disorders in Individuals with Multiple Sclerosis

Neuroticism is a personality trait characterised by a heightened tendency to experience negative emotions such as anxiety, depression, and emotional instability [32]. Research has consistently demonstrated that neuroticism is a key risk factor for the development and maintenance of DE symptoms, as individuals high in neuroticism are more likely to engage in maladaptive coping mechanisms, including restrictive eating, binge eating, and purging behaviours [5]. A large body of the literature has established a link between neuroticism and DE symptoms in community [7] and inpatient and outpatient ED populations [5].

Individuals with MS often also exhibit elevated levels of neuroticism compared to the general population, potentially due to the unpredictable and progressive nature of the disease [33,34]. The psychological distress associated with MS, including uncertainty about disease progression, functional limitations, and social isolation, may amplify the impact of neuroticism on maladaptive eating behaviours. Given that neuroticism is linked to heightened sensitivity to stress and negative self-perception [35,36], individuals with MS who score high on this trait may be particularly susceptible to developing low self-esteem, which in turn could lead to body dissatisfaction and DE behaviours. However, no studies have examined the role of neuroticism and its relationship with DE symptoms in individuals with MS, representing a significant gap in the literature.

### 1.3. The Mediating Role of Self-Esteem and Body Dissatisfaction

Self-esteem, defined as an individual’s overall sense of self-worth, is an important psychological factor in the development of EDs [37]. Low self-esteem has been identified as a key contributor to the onset and maintenance of DE symptoms, as individuals with poor self-worth are more likely to engage in maladaptive eating behaviours as a means of coping with negative emotions [38,39,40]. Research suggests that self-esteem plays a crucial mediating role in the relationship between body dissatisfaction and DE, with additional influences from depression and negative affect. Brechan et al. [39] found that the effect of body dissatisfaction on DE was fully mediated by self-esteem and depression. Their findings indicate that self-esteem and depression are more proximal factors in predicting DE than body dissatisfaction itself. Similarly, Cruz-Sáez et al. [40] demonstrated that self-esteem and negative affect sequentially mediated the relationship between body dissatisfaction and DE, with self-esteem serving as a particularly strong mediator among boys. Additionally, Murray et al. [38] provided longitudinal evidence that stress indirectly predicted body dissatisfaction through reductions in self-esteem and heightened body image importance, further reinforcing the role of self-esteem as a central mechanism linking negative emotional states to body dissatisfaction. Collectively, these studies suggest that interventions targeting self-esteem may be particularly effective in mitigating the impact of body dissatisfaction on DE, potentially offering more sustainable benefits than interventions focused solely on body image concerns.

Previous research has shown that individuals with MS also report lower self-esteem compared to controls [41], and this reduction in self-worth is often linked to increased depressive symptoms and poorer quality of life [42]. Given the well-established relationship between low self-esteem and DE [38,39,40], it is plausible that individuals with MS who experience diminished self-esteem may be at heightened risk for engaging in DE behaviours as a means of coping with perceived inadequacies. However, no studies have directly investigated the role of self-esteem in mediating the relationship between neuroticism and DE in MS, marking another significant gap in the literature. The present study seeks to fill this gap by investigating whether neuroticism contributes to DE symptoms through self-esteem and body dissatisfaction, thereby offering a more comprehensive understanding of the psychological mechanisms involved.

### 1.4. The Present Study

**Figure 1:**
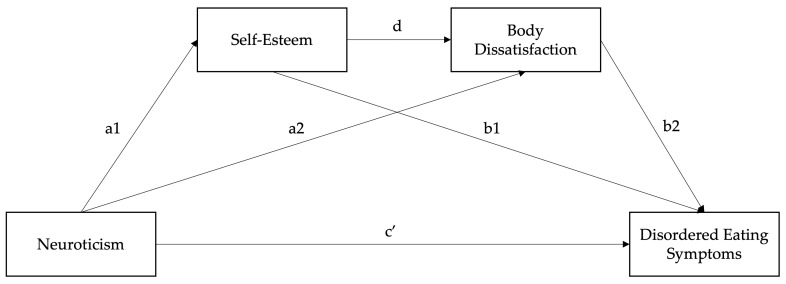
Path diagram of the proposed serial mediation relationship.

Existing studies have found that lower levels of self-esteem and higher levels of body dissatisfaction significantly mediate the relationship between personality, namely neuroticism, and DE in individuals with EDs [13,14,15]. However, these relationships have been unexplored in individuals with MS. Hence, the aim of the current study was to investigate the mediating roles of self-esteem and body dissatisfaction in the relationship between neuroticism and DE in individuals with MS. Specifically, we hypothesised that self-esteem and body dissatisfaction would, respectively, and serially mediate the relationship between neuroticism and DE in people with MS. Based on the past literature, we expected that neuroticism would be positively associated with DE symptoms (total effect c), such that higher neuroticism is linked to greater DE symptoms [5,6,7,8]. As illustrated in Figure 1, we hypothesised that neuroticism would be associated with lower self-esteem (a1) [11,12] and higher body dissatisfaction (a2) [9,10], both of which would be linked to more severe DE (b1 and b2) [14,15]. In addition, we hypothesised that neuroticism would be associated with lower self-esteem (a1) [11,12], which in turn would be linked to increased body dissatisfaction (d) [13,38,39,40], ultimately leading to more severe DE (b2) [39,40].

## 2. Materials and Methods

### 2.1. Participants and Procedure

Participants were recruited from 2019 to 2023 as part of a research programme examining transdiagnostic psychological mechanisms underlying depression, anxiety, and EDs in community, medically ill, and psychological help-seeking populations. Participants were recruited using online advertisements on peer support Facebook groups, websites, and forums; newsletters of relevant organisations and support services (e.g., MS Australia); and advertisements posted on the Royal Melbourne Hospital noticeboards. Interested participants were invited to complete an online survey. All participants provided informed consent to participate and for their data to be used for publication purposes. This study was approved by The University of Melbourne ethics committee (Project number: 2021-12495-20321-4). Data from a total of 275 participants with MS (*M*_age_ = 43.03 years, 79.3% female, 68.3% relapsing-remitting MS) was included in the final analysis.

### 2.2. Materials

#### 2.2.1. Sociodemographic and Clinical Characteristics

Data on age, gender, ethnicity, country of residence, highest education level, employment, and marital status were collected. Participants were asked to self-report whether they had received a physician-confirmed diagnosis of MS and prior or current diagnosis of depression, anxiety, and EDs. Participants were also asked to report disease-related characteristics, including MS type, duration since MS diagnosis and onset, MS relapse, and whether they were taking disease-modifying medication. Ambulation level was assessed using the Self-Reported Disability Status Scale (SRDSS) [43]. The SRDSS is a self-report proxy measure used to estimate categories of the Expanded Disability Status Scale (EDSS), which is the most commonly used measure of disability in MS [44]. Responses to three mobility-related questions on the SRDSS result in three outcomes, namely, SRDSS < 3.5, 4 to 6.5, and >7, with higher scores indicating greater level of ambulation difficulty. Participants also self-reported their height (centimetres or inches) and weight (kilograms or pounds), based on which BMI was calculated.

#### 2.2.2. Psychological Variables

Neuroticism was assessed using the 44-item Big Five Inventory (BFI) [45]. The BFI-Neuroticism subscale consists of eight items and measures a predisposition towards negative emotions (e.g., depression, anxiety, stress, anger) and emotional instability [46]. Participants responded to statements (e.g., ‘I can be moody’, ‘I am someone who is emotionally stable, not easily upset’) on a five-point Likert scale ranging from 1 (disagree strongly) to 5 (agree strongly). Three items were reverse scored. A total score was created by summing all items, with higher scores indicating higher levels of neuroticism.

Self-esteem was measured using the 10-item Rosenberg Self-esteem Scale (RSES-10) [47]. Participants rated the degree to which they agreed with statements (e.g., ‘I feel that I have a number of good qualities’) on a four-point Likert scale ranging from 0 (strongly disagree) to 3 (strongly agree). Five items were reverse scored so that higher total score corresponded to higher self-esteem.

Body dissatisfaction was examined using the eight-item Body Shape Questionnaire (BSQ-8) [48]. Items reflect concerns about body shape and body dissatisfaction and are rated on a scale from 1 (never) to 6 (always). Higher scores indicate higher body dissatisfaction.

DE symptoms were assessed using the 26-item Eating Attitudes Test (EAT-26) [49]. Participants rated the frequency of attitudes, feelings, and behaviours related to food and eating (e.g., ‘I vomit after I have eaten’, ‘I am preoccupied with a desire to be thinner’) on a six-point Likert scale ranging from 0 (never, sometimes, rarely) to 3 (always). The EAT-26 items form three subscales, including dieting, bulimia and food preoccupation, and oral control. In the current study, the total EAT-26 score was used by summing all items. A total score of 20 or greater indicates a probable ED and requires further diagnostic investigation from a qualified professional [50]. A total score below 20 indicates a low level of concern about dieting, body weight, or problematic eating behaviours.

### 2.3. Statistical Analyses

#### 2.3.1. Data Inspection

All analyses were conducted in R [51]. A total of 309 participants with MS accessed the online survey, with a final sample of 275 participants with MS who completed the questionnaires. All primary variables included in the model were within acceptable limits for skewness and kurtosis (i.e., ±1). The percentage of missing data ranged from 0% to 2.9% across variables, with 101 incomplete cases. Following recommended practices [52,53], missing data were handled using multiple imputations with predictive-mean matching (100 datasets and 20 iterations) via the mice package [54]. Model parameters were estimated in each imputed dataset separately and combined using Rubin’s rule [55]. Sensitivity analysis with complete cases suggested that imputation did not alter path coefficients or significance (see Table A1). There was no evidence of multicollinearity between variables according to the variance inflation factor (VIF) and Pearson bivariate correlations.

#### 2.3.2. Serial Mediation Model

The serial mediation model was fitted using the lavaan [56], lavaan.mi [57], and semTools [58] packages. We specified a path model with no latent construct using sum scores of the BFI-Neuroticism, RSES-10, BSQ-8, and the EAT-26. Therefore, model fit indices were not computed. R-squares for the mediating and dependent variables were reported to assess the robustness of the results. Monte-Carlo confidence intervals based on 50,000 replications were constructed to test the statistical significance of hypothesised pathways. Reported path coefficients are unstandardised. To further explore the single and serial mediating effects of self-esteem and body dissatisfaction, we also compared specific indirect pathways. The current sample size has been considered adequate for a serial mediation analysis using a path modelling approach [59]. Further details of the planned statistical analyses and sample size justification can be found in our registration document: https://osf.io/bamrv, accessed on 17 March 2025.

**Figure A1:**
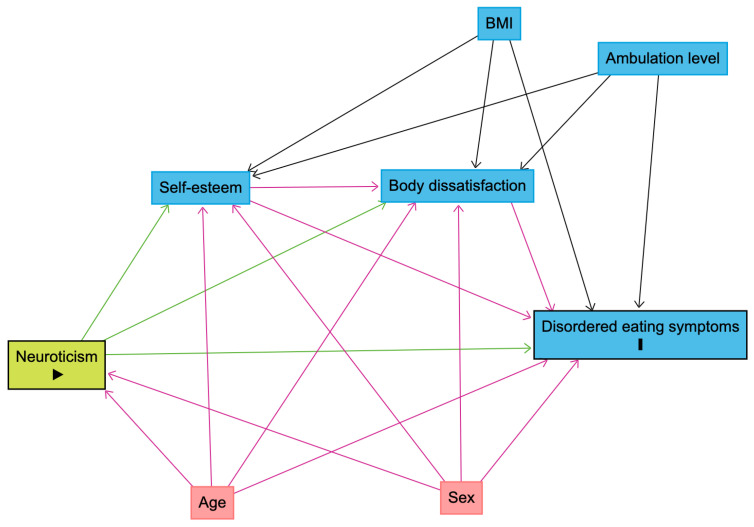
Directed acyclic graph (DAG) representing theoretical associations between neuroticism, self-esteem, body dissatisfaction, DE, and covariates. Green square represents the predictor or exposure. Blue squares represent the outcome (DE symptoms) and ancestors of the outcome (i.e., variables hypothesised to be causal determinants of the outcome). Pink squares represent ancestors of both the predictor and the outcome. Green line represents causal path directly linking the predictor to outcomes. Pink line represents biassing paths.

To identify confounders that need to be controlled for in order to minimise bias when estimating the effect of neuroticism on DE (i.e., the minimum sufficient adjustment set; MSAS), we built a conceptual diagram based on the previous literature. We used the DAGitty software [60] to construct a directed acyclic graph (DAG) (see Figure A1). Potential demographic and disease-related correlates of DE in both ED and MS samples included age [61], sex [14], BMI [2], and level of ambulation [62]. These variables were identified as the MSAS for both total and direct effects and were, thus, included as covariates in the current analyses. Although the DAG method is commonly used in models with causal implications to isolate causal effects from confounded effects, it should be noted that the current study is limited in its ability to assess causality due to the cross-sectional nature of our data.

## 3. Results

### 3.1. Sample Characteristics

Table 1 presents sociodemographic and MS characteristics of the included sample (*N* = 275). Most participants identified as female (79.27%), of Anglo-Celtic background (76.73%), and reported a diagnosis of relapsing-remitting MS (67.27%). The mean age was 43.03 years (SD = 12.88), with ages ranging from 19 to 81 years. Participants reported a mean duration of 8.43 years (SD = 8.13) since MS diagnosis and 12.15 years (SD = 11.11) since MS onset. The mean BMI was 29.13 kg/m^2^ (SD = 11.38).

Previous and current mental health diagnoses are summarised in Table 2. Approximately 21% reported a lifetime diagnosis of ED (*N* = 58/275), with anorexia nervosa being the most common. Among participants diagnosed with ED, 67.24% (*N* = 39/275) reported having a current diagnosis. The total EAT-26 score indicated that 76 (27.64%) participants were at risk of an ED (provided a score of >20). In addition, a small proportion of participants reported having comorbid ED and depression (*N* = 39, 14.18%), ED and anxiety (*N* = 39, 14.18%), or ED, depression, and anxiety (*N* = 33, 12.00%). Nearly half of participants (*N* = 132, 48.00%) reported currently taking anti-depressant or anti-anxiety medication.

### 3.2. Descriptive Statistics and Correlations

Internal reliability, means, standard deviations, and correlations between continuous study variables are reported in Table 3. All variables in the mediation model were significantly correlated and demonstrated good to excellent internal reliability in the present sample (Cronbach’s α = 0.82–0.94).

### 3.3. Serial Mediation Model

**Figure 2:**
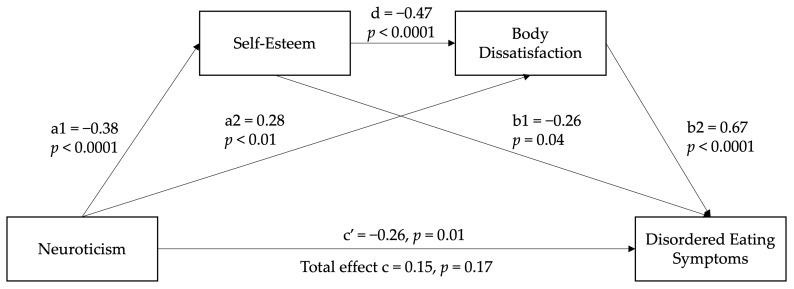
Serial mediation model with pooled path coefficients (*N* = 275). Model path coefficients were combined across 100 multiply imputed datasets using Rubin’s rule. Unstandardised path coefficients are depicted. All pathways were significant.

A serial mediation analysis was conducted to examine the relationship between neuroticism, DE, self-esteem, and body dissatisfaction. Age, gender, BMI, and level of ambulation were included as covariates. Figure 2 presents the pooled unstandardised path coefficients of the hypothesised model across 100 multiply imputed datasets.

Table 4 reports standardised path coefficients and comparisons of specific mediation effects. The total indirect effect of neuroticism on DE symptoms was significant, β = 0.40, SE = 0.08, 95% CI: [0.25, 0.57]. The direct effect of neuroticism on DE symptoms accounting for self-esteem and body dissatisfaction was also significant, β = −0.26, SE = 0.10, 95% CI: [−0.46, −0.05]. However, the total model effect (β = 0.15, SE = 0.11, 95% CI: [−0.07, 0.37]) was not significant. Of note, there was evidence for a serial mediation effect of neuroticism via self-esteem and body dissatisfaction, β = 0.12, SE = 0.04, 95% CI: [0.06, 0.20]. The indirect effects of neuroticism on DE symptoms through self-esteem (β = 0.10, SE = 0.05, 95% CI: [0.01, 0.20]) and body dissatisfaction (β = 0.19, SE = 0.06, 95% CI: [0.06, 0.33]) were significant. Pairwise comparisons indicated no significant differences between the single and serial mediating pathways. The full model accounted for 43.0% of the variance in DE symptoms. The proportion of variance accounted for in self-esteem and body dissatisfaction was 34.6% and 26.4%, respectively.

## 4. Discussion

In the current study, we assessed whether neuroticism would be significantly associated with lower self-esteem, which in turn would be associated with increased body dissatisfaction and related to more severe DE symptoms. The hypothesis that self-esteem and body dissatisfaction play a mediating role in the relationship between neuroticism and DE symptoms in individuals with MS was supported. Our results also showed significant relationships between self-esteem and body dissatisfaction, and both factors were significantly related to DE symptoms in the current MS sample. The hypothesised serial pathway (neuroticism → self-esteem → body dissatisfaction → DE symptoms) was significant, suggesting that the interplay between these two factors is relevant in understanding how neuroticism is linked to DE symptoms.

Consistent with research that has demonstrated significant relationships between neuroticism and lower self-esteem [13,15,16] and neuroticism and increased body dissatisfaction [14] in community and clinical samples with EDs, our results show that these relationships are also significant in people with MS. More specifically, individuals with MS who reported higher levels of neuroticism also experienced lower self-esteem, which may increase body dissatisfaction and, in turn, more DE symptoms. The present study builds upon previous findings by demonstrating that neuroticism is associated with DE symptoms in MS [7,8] and that self-esteem and body dissatisfaction are important factors to consider in understanding DE symptoms in MS [9,10,11,12]. The sequential effects of self-esteem and body dissatisfaction further indicate that low self-esteem is associated with DE symptoms through more body image concerns in people with MS. However, the proposed model only provides one plausible pathway through which neuroticism, self-esteem, body dissatisfaction, and DE symptoms are related. Future longitudinal studies may utilise this model to determine how neuroticism facilitates adverse DE outcomes in people with MS over time.

Interestingly, our findings showed a suppression effect of self-esteem and body dissatisfaction on DE symptoms. That is, while the total indirect effect of neuroticism on DE symptoms was positive, the negative direct effect indicates that higher neuroticism was associated with fewer ED symptoms when holding self-esteem and body dissatisfaction constant. This may be likely due to confounding factors that were not accounted for in our model, such as other personality traits (e.g., conscientiousness [6], extraversion [8], openness to experience [63], and perfectionism [6]) and cognitive vulnerabilities (e.g., intolerance of uncertainty [64], emotion regulation [65], and distress tolerance [66]) that have been robustly linked to both neuroticism and EDs.

It is also possible that the relationship between neuroticism and DE in our MS sample differs from that observed in other populations. Prior research has supported the existence of ‘healthy neuroticism’ [67,68], where individuals with higher levels of neuroticism and conscientiousness report better health outcomes due to increased body vigilance [69], greater likelihood of seeking medical attention [70], and less engagement in maladaptive health behaviours [70,71,72]. For instance, at high levels of conscientiousness, high neuroticism has been associated with lower odds of smoking [71], problematic alcohol consumption [72], lower levels of inflammatory biomarkers [73], and higher levels of physical activity [70,74]. Similarly, recent findings from a community sample of 712 adults suggest that high levels of trait neuroticism and conscientiousness reliably predicted reduced DE behaviours such as less disinhibited eating and hunger and increased self-regulatory eating restraint [75]. In addition, evidence from the broader ED literature has identified mixed associations between neuroticism and DE, particularly when specific facets of neuroticism and specific DE behaviours are considered [7]. For example, the impulsiveness facet of neuroticism reflecting low self-control has been positively associated with binge or emotional eating, compensatory behaviours, and global eating pathology but negatively associated with dietary restraint [7]. Other facets of neuroticism demonstrated a consistent positive association with DE behaviours [7].

Together, these findings suggest that the neuroticism-ED link in MS is complex and nuanced, and results from our study should be taken as the first attempt to explore this relationship. As conscientiousness was not examined in our study, the potential presence and prevalence of healthy neuroticism in people with MS and its relationship to DE behaviours warrant further examination. Future studies may also adopt a facet-level approach to measuring neuroticism and distinguishing between specific DE behaviours associated with different ED phenotypes to further elucidate the effect of neuroticism on DE symptoms in MS.

The current study found that 21% of participants reported a diagnosis of an ED, with anorexia nervosa and bulimia nervosa being the most common. This is higher than the prevalence rates of EDs identified in non-MS samples with 12-month and lifetime rates being up to 2.58% in females [17]. Relatedly, over half of our sample (53.45%) reported a co-morbid depressive disorder, with major depressive disorder being the most common, and nearly half of the sample (47.27%) reported a co-morbid anxiety disorder, with GAD being the most common disorder. Relatedly, nearly half of the current sample (48%) also indicated that they are taking anti-depressant or anti-anxiety medication. This is consistent with previous research that has found high rates of depression and anxiety in individuals with MS [24].

### 4.1. Clinical Implications

Given the study’s findings that self-esteem and body dissatisfaction are significantly related to DE symptoms in individuals with MS, these factors should be considered in models of DE in individuals with MS. Consideration should also be given in integrating routine screening for DE in clinical settings. A practical first step would be to employ additional validated screening tools such as the SCOFF questionnaire [76], which is a five-item self-report measure that can quickly identify individuals at risk of eating disorders. In addition, incorporating brief assessments of all facets of neuroticism, self-esteem, and body dissatisfaction, such as the measures included in the current study, may help identify psychosocial vulnerabilities that contribute to DE symptoms in individuals with MS.

Findings suggest that preventive interventions and psychological support for individuals with MS should not only address mood and physical function but should also consider self-image and eating behaviours. Cognitive–behavioural approaches tailored to body image concerns, self-worth, and coping strategies related to disease progression and physical changes in MS (e.g., [77,78,79]) may be beneficial. Psychoeducation and mindfulness-based interventions targeting body acceptance and self-compassion [80,81,82] may also help reduce the risk of DE symptoms in those with MS. Importantly, interdisciplinary care involving neurologists, psychologists, dietitians, and rehabilitation specialists [83] can foster early detection and a more holistic approach to support individuals with MS who may be vulnerable to DE.

Finally, mental health professionals should also consider screening for co-occurring depressive and anxiety symptoms, given the high prevalence of these symptoms in the current MS sample. Targeted interventions that address this comorbid presentation are crucial, as the combination of DE [84], body dissatisfaction [79], low self-esteem [23], and affective symptoms [85] may compound distress and interfere with disease management and quality of life in those affected by MS. Multimodal psychological approaches that integrate mood regulation, body image work, and self-esteem enhancement may be particularly effective in supporting individuals with MS who present with overlapping challenges.

### 4.2. Limitations and Future Directions

There were several limitations to the current study. The serial mediation model in the current study was examined using cross-sectional data. It is recommended that future studies employ a longitudinal design, such as cross-lagged panel models, to facilitate accurate inferences about the presence, strength, and direction of causal relationships [86]. The current study used self-report measures rather than structured clinical interviews undertaken by a mental health professional for symptoms and diagnoses of depression, anxiety, and EDs, which could affect the accuracy of the findings, including possible social desirability, recall, or under-diagnosis biases. MS diagnosis was also self-reported. However, participants were recruited from a large MS centre, online MS groups, and through MS organisations. Future work should confirm the participant MS diagnosis with their neurologist.

The current study did not differentiate between specific DE symptoms. As a result, it remains unclear whether individuals with MS were more likely to engage in restrictive eating, binge eating, or compensatory behaviours such as purging. Additionally, facets of neuroticism were also not examined. Future studies should adopt a more granular analysis of specific DE symptom profiles and facets of neuroticism to better understand their relationships and to tailor assessments and interventions focusing on EDs. Future research should also explore whether the observed relationships vary across MS subtypes (relapse remitting, primary progressive), which we were not able to examine due to our sample size. This could provide clinically relevant information for tailoring interventions to different MS disease presentations. Lastly, the included sample was predominantly female (79.3%). Although gender was controlled for in our analyses, previous findings that women living with MS reported higher body dissatisfaction [27] and lower self-esteem [2] in comparison to men suggest that this gender imbalance could have influenced the results. Future studies are encouraged to recruit and focus on these relationships in males with MS.

## 5. Conclusions

This study represents the first attempt to explore neuroticism, self-esteem, and body dissatisfaction as correlates of DE symptoms in a sample of people with MS. The existing mediating roles of self-esteem and body dissatisfaction in the relationship between neuroticism and DE symptoms identified in community and ED samples appear to extend to people with MS. Our findings suggest that self-esteem and body dissatisfaction should be considered in models explaining the development and maintenance of EDs in individuals with MS. Targeting self-esteem and body dissatisfaction may be relevant for treating and preventing DE symptoms in individuals with MS who have high levels of neuroticism, although longitudinal research is needed to examine causal pathways.

## Figures and Tables

**Table 1:** Sociodemographic and MS-related characteristics of the included sample (*N* = 275).

Demographic	*N* (%)
Gender	
Male	55 (20.0%)
Female	218 (79.3%)
Non-binary	2 (<1.0%)
Ethnicity	
Anglo-Celtic	211 (76.7%)
Asian (Eastern, Southern, Southeastern)	8 (2.9%)
Indigenous Australian and/or Torres Strait Islander	12 (4.4%)
Hispanic or Latin American	4 (1.5%)
Middle Eastern	3 (1.1%)
African American	22 (8.0%)
Other	15 (5.5%)
Country of current residence	
Australia	145 (52.7%)
New Zealand	6 (2.2%)
UK	14 (5.1%)
USA	88 (32.0%)
Other	22 (8.0%)
Highest level of education	
Postgraduate	51 (18.6%)
Bachelor’s degree	76 (27.6%)
Year 12 (high school) or equivalent	38 (13.8%)
Diploma or certificate level	103 (37.5%)
Below high school	7 (2.6%)
Relationship status	
Married	173 (62.9%)
Partnered/De facto	13 (4.7%)
Single/Never married	38 (13.8%)
Divorced/Widowed/Separated	51 (18.6%)
Employment status	
Full-time	110 (40.0%)
Part-time/Casual	73 (26.6%)
Unemployed	92 (33.5%)
MS type	
Relapsing-remitting	185 (68.3%)
Progressive (primary or secondary)	80 (29.1%)
Other/Not sure	10 (3.6%)
MS relapses in the past 12 months	
None	129 (46.9%)
1 to 3	119 (43.3%)
More than 3	27 (9.8%)
Current MS relapse at the time of survey	
Yes	75 (27.3%)
No	200 (72.7%)
Current disease-modifying treatment/s	
Yes	185 (67.3%)
No	90 (32.7%)
Level of ambulation	
SRDSS < 3.5	117 (42.5%)
SRDSS 4 to 6.5	94 (34.2%)
SRDSS > 7	18 (6.6%)
Missing	46 (16.7%)

**Table 2:** Self-reported depression, anxiety, and eating disorder diagnoses (*N* = 275).

Diagnosis	*N* (% Sample)
Depressive disorder diagnosis	
Total	147 (53.5%)
Current	104 (37.8%)
Recovered/lifetime	43 (15.6%)
Anxiety disorder diagnosis	
Total	130 (47.3%)
Current	114 (41.5%)
Recovered/lifetime	16 (5.8%)
Eating disorder diagnosis	
Total	58 (21.1%)
Current	39 (14.2%)
Recovered/lifetime	19 (6.9%)
Depressive disorder type ^a^	
Major depressive disorder (incl. post-natal depression)	52 (18.9%)
Persistent depressive disorder	42 (10.4%)
Premenstrual dysphoric disorder	7 (1.7%)
Not sure/other	55 (13.6%)
Anxiety disorder type ^a^	
Generalised anxiety disorder (GAD)	73 (26.6%)
Panic disorder	16 (5.8%)
Agoraphobia	3 (1.1%)
Specific phobia	7 (2.6%)
Social anxiety disorder	33 (12.0%)
Health/illness anxiety	7 (2.6%)
Separation anxiety disorder	13 (4.7%)
Not sure/other	10 (3.6%)
Eating disorder type ^a^	
Anorexia nervosa (restricting and binge/purging)	34 (12.4%)
Bulimia nervosa (purging and non-purging)	26 (9.5%)
Binge eating disorder	4 (1.5%)
Currently taking antidepressant or anti-anxiety medication	
Yes	132 (48.0%)
No	143 (52.0%)

^a^ Participants could report more than one type of disorder. Participants answered the question: “Have you had a diagnosis of a depressive, anxiety, or eating disorder given to you by a health professional?”.

**Table 3:** Means, standard deviations, and Pearson correlations between study variables.

Variables	Cronbach’s α	*M*	*SD*	1	2	3	4	5	6	7
1. Neuroticism (BFI)	0.82	24.87	6.56	–						
2. Self-esteem (RSES)	0.87	13.87	5.69	−0.50 **	–					
3. Body dissatisfaction (BSQ)	0.90	24.73	9.55	0.37 **	−0.43 **	–				
4. DE symptoms (EAT-26)	0.94	13.86	11.22	0.14 *	−0.37 **	0.57 **	–			
5. Age (years)	-	43.03	12.88	−0.18 **	0.40 **	−0.23 **	−0.36 **	–		
6. BMI	-	29.13	11.38	0.00	−0.13	0.19 **	−0.06	−0.01	–	
7. Ambulation level (SRDSS)	-	-	-	−0.09	0.03	0.01	−0.09	0.25 **	0.02	–

Note. All correlations are two-tailed. * *p* < 0.05. ** *p* < 0.01. *M* = mean. *SD* = standard deviation. BFI = Big Five Inventory. RSES = Rosenberg Self-esteem Scale (10 items). BSQ = Body Shape Questionnaire (8 items). EAT-26 = Eating Attitudes Test (26 items). SRDSS = Self-Report Disability Status Scale (3 items).

**Table 4:** Standardised and unstandardised path coefficients of the serial mediation model controlling for age, gender, BMI, and ambulation level.

Model Pathway	β	SE	t	*p*	LL 95% CI	UL 95% CI
Neuroticism → Self-esteem (a1)	−0.38 (−0.44)	0.05	−7.88	<0.001	−0.01	0.20
Neuroticism → Body dissatisfaction (a2)	0.28 (0.19)	0.10	2.83	0.005	**0.09**	**0.47**
Self-esteem → DE symptoms (b1)	−0.26 (−0.13)	0.13	−2.06	0.039	**−0.46**	**−0.05**
Body dissatisfaction → DE symptoms (b2)	0.67 (0.57)	0.07	9.76	<0.001	**0.53**	**0.80**
Self-esteem → Body dissatisfaction (d)	−0.47 (−0.27)	0.12	−3.90	<0.001	**−0.70**	**−0.23**
Total model effect	0.15 (0.09)	0.11	1.36	0.173	−0.07	0.37
Direct effect (c’)	−0.26 (−0.15)	0.10	−2.49	0.013	**−0.46**	**−0.05**
Total indirect effect	0.40 (0.22)	0.08	5.06	<0.001	**0.25**	**0.57**
Neuroticism → Self-esteem → DE symptoms (Ind1)	0.10 (0.06)	0.05	1.98	0.048	**0.01**	**0.20**
Neuroticism → Body dissatisfaction → DE symptoms (Ind2)	0.18 (0.11)	0.07	2.74	0.006	**0.06**	**0.33**
Neuroticism → Self-esteem → Body dissatisfaction → DE symptoms (Ind3)	0.12 (0.07)	0.04	2.99	0.003	**0.05**	**0.20**
Pairwise comparisons of indirect effects						
Ind1–Ind2	−0.09 (−0.05)	0.09	−0.98	0.326	−0.26	0.09
Ind1–Ind3	−0.02 (−0.01)	0.06	−0.29	0.774	−0.15	0.11
Ind2–Ind3	0.07 (0.04)	0.09	0.77	0.440	−0.10	0.24

Note. *N* = 275. Significant pathways are noted in bold (95% confidence interval does not cross zero). 95% confidence intervals around estimates of unstandardised effects were obtained from Monte-Carlo simulations with 50,000 replications. Unstandardised effects are shown outside parentheses. Standardised effects are shown inside parentheses. Ind1–Ind2 compares the single mediating effects of self-esteem and body dissatisfaction. Ind1–Ind3 compares the single mediating effect of self-esteem to the serial mediating effect through both mediators. Ind2–Ind3 compares the single mediating effect of body dissatisfaction to the serial mediating effect.

## Data Availability

De-identified data related to the results of this study can be requested from the corresponding author upon reasonable request. A data sharing agreement will be developed with the corresponding author and researchers.

## Abbreviations

The following abbreviations are used in this manuscript:

MS	Multiple sclerosis
ED	Eating disorder
DE	Disordered eating
BFI	Big Five Inventory
RSES	Rosenberg Self-esteem Scale
BSQ	Body Shape Questionnaire
EAT-26	Eating Attitudes Test

## Appendix A

**Table A1:** Standardised and unstandardised path coefficients of the serial mediation model controlling for age, gender, BMI, and ambulation level (complete case analysis).

Model Pathway	β	SE	z	*p*	LL 95% CI	UL 95% CI
Neuroticism → Self-esteem (a1)	−0.42 (−0.46)	0.06	−7.17	<0.001	−0.54	−0.31
Neuroticism → Body dissatisfaction (a2)	0.23 (0.15)	0.12	1.98	0.048	**0.00**	**0.45**
Self-esteem → ED symptoms (b1)	−0.27 (−0.14)	0.13	−2.00	0.046	**−0.53**	**−0.01**
Body dissatisfaction → ED symptoms (b2)	0.68 (0.59)	0.08	9.05	<0.001	**0.53**	**0.82**
Self-esteem → Body dissatisfaction (d)	−0.44 (−0.27)	0.13	−3.36	0.001	**−0.70**	**−0.18**
Total model effect	0.16 (0.09)	0.13	1.29	0.198	−0.09	0.41
Direct effect (c’)	−0.23 (−0.13)	0.12	−2.00	0.045	**−0.45**	**−0.00**
Total indirect effect	0.39 (0.23)	0.10	4.12	<0.001	**0.21**	**0.58**
Neuroticism → Self-esteem → ED symptoms (ind1)	0.11 (0.07)	0.06	1.92	0.054	**0.00**	**0.23**
Neuroticism → Body dissatisfaction → ED symptoms (ind2)	0.15 (0.09)	0.08	1.94	0.053	**0.00**	**0.32**
Neuroticism → Self-esteem → Body dissatisfaction → ED symptoms (ind3)	0.13 (0.07)	0.04	2.89	0.053	**0.05**	**0.22**
Pairwise comparisons of indirect effects						
Ind1–Ind2	−0.04 (−0.03)	0.10	−0.42	0.673	−0.25	0.16
Ind1–Ind3	−0.01 (−0.01)	0.07	−0.19	0.851	−0.16	0.13
Ind2–Ind3	0.03 (0.02)	0.10	0.28	0.777	−0.18	0.23

Note. *N* = 174. Significant pathways are noted in bold (95% confidence interval does not cross zero). 95% confidence intervals around estimates of unstandardised effects were obtained from Monte-Carlo simulations with 50,000 replications. Unstandardised effects are shown outside parentheses. Standardised effects are shown inside parentheses. The full model accounted for 45.5% of the variance in ED symptoms. The proportion of variance accounted for in self-esteem and body dissatisfaction was 35.0% and 25.5%, respectively.

## References

[1] Ward M., Goldman M.D. (2022). Epidemiology and pathophysiology of multiple sclerosis. Continuum.

[2] Stevens S.D., Thompson N.R., Sullivan A.B. (2019). Prevalence and correlates of body image dissatisfaction in patients with multiple sclerosis. Int. J. MS Care.

[3] Glasofer D.R., Attia E., Pike K.M., Castonguay L.G., Oltmanns T.F. (2013). Eating disorders. Psychopathology: From Science to Clinical Practice.

[4] Crone C., Fochtmann L.J., Attia E., Boland R., Escobar J., Fornari V., Golden N., Guarda A., Jackson-Triche M., Manzo L. (2023). The american psychiatric association practice guideline for the treatment of patients with eating disorders. Am. J. Psychiatry.

[5] Cassin S.E., von Ranson K.M. (2005). Personality and eating disorders: A decade in review. Clin. Psychol. Rev..

[6] Farstad S.M., McGeown L.M., von Ranson K.M. (2016). Eating disorders and personality, 2004–2016: A systematic review and meta-analysis. Clin. Psychol. Rev..

[7] Gilmartin T., Gurvich C., Sharp G. (2022). The relationship between disordered eating behaviour and the five factor model personality dimensions: A systematic review. J. Clin. Psychol..

[8] Miller J.L., Schmidt L.A., Vaillancourt T., McDougall P., Laliberte M. (2006). Neuroticism and introversion: A risky combination for disordered eating among a non-clinical sample of undergraduate women. Eat. Behav..

[9] Allen M.S., Robson D.A. (2020). Personality and body dissatisfaction: An updated systematic review with meta-analysis. Body Image.

[10] Swami V., Taylor R., Carvalho C. (2011). Body dissatisfaction assessed by the photographic figure rating scale is associated with sociocultural, personality, and media influences. Scand. J. Psychol..

[11] Clague C.A., Prnjak K., Mitchison D. (2023). “I don’t want them to judge me”: Separating out the role of fear of negative evaluation, neuroticism, and low self-esteem in eating disorders. Eat. Behav..

[12] Jones H., McIntosh V.V.W., Britt E., Carter J.D., Jordan J., Bulik C.M. (2022). The effect of temperament and character on body dissatisfaction in women with bulimia nervosa: The role of low self-esteem and depression. Eur. Eat. Disord. Rev..

[13] Skorek M., Song A.V., Dunham Y. (2014). Self-esteem as a mediator between personality traits and body esteem: Path analyses across gender and race/ethnicity. PLoS ONE.

[14] MacNeill L.P., Best L.A., Davis L.L. (2017). The role of personality in body image dissatisfaction and disordered eating: Discrepancies between men and women. J. Eat. Disord..

[15] Cervera S., Lahortiga F., Martinez-Gonzalez M.A., Gual P., de Irala-Estevez J., Alonso Y. (2003). Neuroticism and low self-esteem as risk factors for incident eating disorders in a prospective cohort study. Int. J. Eat. Disord..

[16] Krauss S., Dapp L.C., Orth U. (2023). The link between low self-esteem and eating disorders: A meta-analysis of longitudinal studies. Clin. Psychol. Sci..

[17] Qian J., Wu Y., Liu F., Zhu Y., Jin H., Zhang H., Wan Y., Li C., Yu D. (2022). An update on the prevalence of eating disorders in the general population: A systematic review and meta-analysis. Eat. Weight. Disord..

[18] Galmiche M., Dechelotte P., Lambert G., Tavolacci M.P. (2019). Prevalence of eating disorders over the 2000-2018 period: A systematic literature review. Am. J. Clin. Nutr..

[19] Leon L., Clemente D., Heredia C., Abasolo L. (2024). Self-esteem, self-concept, and body image of young people with rheumatic and musculoskeletal diseases: A systematic literature review. Semin. Arthritis Rheum..

[20] Bahrami M., Mohamadirizi M., Mohamadirizi S., Hosseini S.A. (2017). Evaluation of body image in cancer patients and its association with clinical variables. J. Educ. Health Promot..

[21] Troncone A., Cascella C., Chianese A., Zanfardino A., Piscopo A., Borriello A., Casaburo F., Del Giudice E.M., Iafusco D. (2020). Body image problems and disordered eating behaviors in Italian adolescents with and without type 1 diabetes: An examination with a gender-specific body image measure. Front. Psychol..

[22] Gascoyne C.R., Simpson S., Chen J., van der Mei I., Marck C.H. (2019). Modifiable factors associated with depression and anxiety in multiple sclerosis. Acta Neurol. Scand..

[23] Mikula P., Timkova V., Fedicova M., Szilasiova J., Nagyova I. (2021). Self-management, self-esteem and their associations with psychological well-being in people with multiple sclerosis. Mult. Scler. Relat. Disord..

[24] Kiropoulos L.A., Kilpatrick T., Holmes A., Threader J. (2016). A pilot randomized controlled trial of a tailored cognitive behavioural therapy based intervention for depressive symptoms in those newly diagnosed with multiple sclerosis. BMC Psychiatry.

[25] Reininghaus E., Reininghaus B., Fitz W., Hecht K., Bonelli R.M. (2012). Sexual behavior, body image, and partnership in chronic illness: A comparison of Huntington’s disease and multiple sclerosis. J. Nerv. Ment. Dis..

[26] Bailey K.A., Dagenais M., Gammage K.L. (2021). Is a picture worth a thousand words? Using photo-elicitation to study body image in middle-to-older age women with and without multiple sclerosis. Qual. Health Res..

[27] Pfaffenberger N., Gutweniger S., Kopp M., Seeber B., Sturz K., Berger T., Gunther V. (2011). Impaired body image in patients with multiple sclerosis. Acta Neurol. Scand..

[28] Matusik E., Durmala J., Ksciuk B., Matusik P. (2022). Body composition in multiple sclerosis patients and its relationship to the disability level, disease duration and glucocorticoid therapy. Nutrients.

[29] Taul-Madsen L., Connolly L., Dennett R., Freeman J., Dalgas U., Hvid L.G. (2021). Is aerobic or resistance training the most effective exercise modality for improving lower extremity physical function and perceived fatigue in people with multiple sclerosis? A systematic review and meta-analysis. Arch. Phys. Med. Rehabil..

[30] Jagannath V.A., Filippini G., Di Pietrantonj C., Asokan G.V., Robak E.W., Whamond L., Robinson S.A. (2018). Vitamin D for the management of multiple sclerosis. Cochrane Database Syst. Rev..

[31] Parks N.E., Jackson-Tarlton C.S., Vacchi L., Merdad R., Johnston B.C. (2020). Dietary interventions for multiple sclerosis-related outcomes. Cochrane Database Syst. Rev..

[32] McCrae R.R., Costa P.T. (2011). The NEO Personality Inventory: Using the five-factor model in counseling. J. Couns. Dev..

[33] Roy S., Drake A.S., Eizaguirre M.B., Zivadinov R., Weinstock-Guttman B., Chapman B.P., Benedict R.H. (2018). Trait neuroticism, extraversion, and conscientiousness in multiple sclerosis: Link to cognitive impairment?. Mult. Scler..

[34] Maggio M.G., Cuzzola M.F., Latella D., Impellizzeri F., Todaro A., Rao G., Manuli A., Calabro R.S. (2020). How personality traits affect functional outcomes in patients with multiple sclerosis: A scoping review on a poorly understood topic. Mult. Scler. Relat. Disord..

[35] Thomson W. (2016). Depression, neuroticism, and the discrepancy between actual and ideal self-perception. Pers. Individ. Dif..

[36] Uliaszek A.A., Zinbarg R.E., Mineka S., Craske M.G., Sutton J.M., Griffith J.W., Rose R., Waters A., Hammen C. (2010). The role of neuroticism and extraversion in the stress-anxiety and stress-depression relationships. Anxiety Stress. Coping.

[37] Fairburn C.G., Cooper Z., Shafran R. (2003). Cognitive behaviour therapy for eating disorders: A “transdiagnostic” theory and treatment. Behav. Res. Ther..

[38] Murray K., Rieger E., Byrne D. (2013). A longitudinal investigation of the mediating role of self-esteem and body importance in the relationship between stress and body dissatisfaction in adolescent females and males. Body Image.

[39] Brechan I., Kvalem I.L. (2015). Relationship between body dissatisfaction and disordered eating: Mediating role of self-esteem and depression. Eat. Behav..

[40] Cruz-Saez S., Pascual A., Wlodarczyk A., Echeburua E. (2020). The effect of body dissatisfaction on disordered eating: The mediating role of self-esteem and negative affect in male and female adolescents. J. Health Psychol..

[41] McCabe M.P. (2005). Mood and self-esteem of persons with multiple sclerosis following an exacerbation. J. Psychosom. Res..

[42] Mikula P., Nagyova I., Krokavcova M., Vitkova M., Rosenberger J., Szilasiova J., Gdovinova Z., Stewart R.E., Groothoff J.W., van Dijk J.P. (2017). Self-esteem, social participation, and quality of life in patients with multiple sclerosis. J. Health Psychol..

[43] Kaufmann M., Salmen A., Barin L., Puhan M.A., Calabrese P., Kamm C.P., Gobbi C., Kuhle J., Manjaly Z.M., Ajdacic-Gross V. (2020). Development and validation of the Self-reported Disability Status Scale (SRDSS) to estimate EDSS-categories. Mult. Scler. Relat. Disord..

[44] Kurtzke J.F. (1983). Rating neurologic impairment in multiple sclerosis: An Expanded Disability Status Scale (EDSS). Neurology.

[45] John O.P., Srivastava S. (1999). The Big-Five trait taxonomy: History, measurement, and theoretical perspectives. Handbook of Personality: Theory and Research.

[46] Costa Mastrascusa R., de Oliveira Fenili Antunes M.L., de Albuquerque N.S., Virissimo S.L., Foletto Moura M., Vieira Marques Motta B., de Lara Machado W., Moret-Tatay C., Quarti Irigaray T. (2023). Evaluating the complete (44-item), short (20-item) and ultra-short (10-item) versions of the Big Five Inventory (BFI) in the Brazilian population. Sci. Rep..

[47] Rosenberg M. Rosenberg Self-Esteem Scale (RSES). https://www.apa.org/obesity-guideline/rosenberg-self-esteem.pdf.

[48] Cooper P., Taylor M., Cooper Z., Fairburn C.G. (1987). The development and validation of the Body Shape Questionnaire. Int. J. Eat. Disord..

[49] Garner D.M., Olmsted M.P., Bohr Y., Garfinkel P.E. (1982). The Eating Attitudes Test: Psychometric features and clinical correlates. Psychol. Med..

[50] Papini N.M., Jung M., Cook A., Lopez N.V., Ptomey L.T., Herrmann S.D., Kang M. (2022). Psychometric properties of the 26-item Eating Attitudes Test (EAT-26): An application of Rasch analysis. J. Eat. Disord..

[51] R Core Team (2024). R: A Language and Environment for Statistical Computing.

[52] Schoemann A.M., Moore E.W.G., Yagiz G. (2025). How and why to follow best practices for testing mediation models with missing data. Int. J. Psychol..

[53] Austin P.C., White I.R., Lee D.S., van Buuren S. (2021). Missing data in clinical research: A tutorial on multiple imputation. Can. J. Cardiol..

[54] Buuren S.v., Groothuis-Oudshoorn K. (2011). Mice: Multivariate imputation by chained equations in R. J. Stat. Softw..

[55] Rubin D.B. (1987). Multiple Imputation for Nonresponse in Surveys.

[56] Rosseel Y. (2012). Lavaan: An R package for structural equation modeling. J. Stat. Softw..

[57] Jorgensen T. (2025). Lavaan.Mi: Fit Structural Equation Models to Multiply Imputed Data. https://CRAN.R-project.org/package=lavaan.mi.

[58] Jorgensen T., Pornprasertmanit S., Schoemann A.M., Rosseel Y. (2025). SemTools: Useful Tools for Structural Equation Modeling. https://CRAN.R-project.org/package=semTools.

[59] Sim M., Kim S.Y., Suh Y. (2022). Sample size requirements for simple and complex mediation models. Educ. Psychol. Meas..

[60] Textor J., van der Zander B., Gilthorpe M.S., Liskiewicz M., Ellison G.T. (2016). Robust causal inference using directed acyclic graphs: The R package ‘dagitty’. Int. J. Epidemiol..

[61] Rohde P., Stice E., Shaw H., Gau J.M., Ohls O.C. (2017). Age effects in eating disorder baseline risk factors and prevention intervention effects. Int. J. Eat. Disord..

[62] Lo Buono V., Bonanno L., Corallo F., Cardile D., D’Aleo G., Rifici C., Sessa E., Quartarone A., De Cola M.C. (2023). The relationship between body image, disability and mental health in patients with multiple sclerosis. J. Clin. Med..

[63] Claes L., Vandereycken W., Luyten P., Soenens B., Pieters G., Vertommen H. (2006). Personality prototypes in eating disorders based on the big five model. J. Pers. Disord..

[64] Clarke E., Kiropoulos L.A. (2021). Mediating the relationship between neuroticism and depressive, anxiety and eating disorder symptoms: The role of intolerance of uncertainty and cognitive flexibility. J. Affect. Disord. Rep..

[65] Dingemans A., Danner U., Parks M. (2017). Emotion regulation in binge eating disorder: A review. Nutrients.

[66] Mattingley S., Youssef G.J., Manning V., Graeme L., Hall K. (2022). Distress tolerance across substance use, eating, and borderline personality disorders: A meta-analysis. J. Affect. Disord..

[67] Friedman H.S. (2000). Long-term relations of personality and health: Dynamisms, mechanisms, tropisms. J. Pers..

[68] Friedman H.S. (2019). Neuroticism and health as individuals age. Pers. Disord..

[69] Weston S.J., Jackson J.J. (2018). The role of vigilance in the relationship between neuroticism and health: A registered report. J. Res. Pers..

[70] Graham E.K., Weston S.J., Turiano N.A., Aschwanden D., Booth T., Harrison F., James B.D., Lewis N.A., Makkar S.R., Mueller S. (2020). Is healthy neuroticism associated with health behaviors? A coordinated integrative data analysis. Collabra Psychol..

[71] Weston S.J., Jackson J.J. (2015). Identification of the healthy neurotic: Personality traits predict smoking after disease onset. J. Res. Pers..

[72] Turiano N.A., Whiteman S.D., Hampson S.E., Roberts B.W., Mroczek D.K. (2012). Personality and substance use in midlife: Conscientiousness as a moderator and the effects of trait change. J. Res. Pers..

[73] Turiano N.A., Mroczek D.K., Moynihan J., Chapman B.P. (2013). Big 5 personality traits and interleukin-6: Evidence for “healthy neuroticism” in a US population sample. Brain Behav. Immun..

[74] Stieger M., Robinson S.A., Bisson A.N., Lachman M.E. (2020). The relationship of personality and behavior change in a physical activity intervention: The role of conscientiousness and healthy neuroticism. Pers. Individ. Dif..

[75] Arend I., Yuen K. (2025). Association between healthy neuroticism and eating behavior as revealed by the NKI Rockland sample. Sci. Rep..

[76] Morgan J.F., Reid F., Lacey J.H. (2000). The SCOFF questionnaire: A new screening tool for eating disorders. West. J. Med..

[77] Tesar N., Baumhackl U., Kopp M., Gunther V. (2003). Effects of psychological group therapy in patients with multiple sclerosis. Acta Neurol. Scand..

[78] Paolucci T., de Sire A., Agostini F., Bernetti A., Salome A., Altieri M., Di Piero V., Ammendolia A., Mangone M., Paoloni M. (2022). Efficacy of interoceptive and embodied rehabilitative training protocol in patients with mild multiple sclerosis: A randomized controlled trial. Front. Neurol..

[79] McCormack D., O’Keeffe D.F., Seery C., Eccles D.F. (2025). The association between body image and psychological outcomes in multiple sclerosis. A systematic review. Mult. Scler. Relat. Disord..

[80] Bogosian A., Hughes A., Norton S., Silber E., Moss-Morris R. (2016). Potential treatment mechanisms in a mindfulness-based intervention for people with progressive multiple sclerosis. Br. J. Health Psychol..

[81] Simpson R., Mair F.S., Mercer S.W. (2017). Mindfulness-based stress reduction for people with multiple sclerosis—A feasibility randomised controlled trial. BMC Neurol..

[82] Hocaloski S., Elliott S., Brotto L.A., Breckon E., McBride K. (2016). A mindfulness psychoeducational group intervention targeting sexual adjustment for women with multiple sclerosis and spinal cord injury: A pilot study. Sex. Disabil..

[83] Wills O., Probst Y., Haartsen J., McMahon A.T. (2024). The role of multidisciplinary MS care teams in supporting lifestyle behaviour changes to optimise brain health among people living with MS: A qualitative exploration of clinician perspectives. Health Expect..

[84] Saul A., Taylor B.V., Blizzard L., Simpson-Yap S., Oddy W.H., Probst Y.C., Black L.J., Ponsonby A.L., Broadley S.A., Lechner-Scott J. (2022). Associations between diet quality and depression, anxiety, and fatigue in multiple sclerosis. Mult. Scler. Relat. Disord..

[85] Hanna M., Strober L.B. (2020). Anxiety and depression in multiple sclerosis (MS): Antecedents, consequences, and differential impact on well-being and quality of life. Mult. Scler. Relat. Disord..

[86] Hamaker E.L., Kuiper R.M., Grasman R.P. (2015). A critique of the cross-lagged panel model. Psychol. Methods.

